# Munc13-1 Translocates to the Plasma Membrane in a Doc2B- and Calcium-Dependent Manner

**DOI:** 10.3389/fendo.2013.00119

**Published:** 2013-09-17

**Authors:** Reut Friedrich, Irit Gottfried, Uri Ashery

**Affiliations:** ^1^Department of Neurobiology, Wise Faculty of Life Sciences, Tel Aviv University, Tel Aviv, Israel; ^2^Sagol School of Neuroscience, Tel Aviv University, Tel Aviv, Israel

**Keywords:** Munc13, Doc2B, calcium, translocation, phorbol ester

## Abstract

Munc13-1 is a presynaptic protein activated by calcium, calmodulin, and diacylglycerols (DAG) that is known to enhance vesicle priming. Doc2B is another presynaptic protein that translocates to the plasma membrane (PM) upon elevation of internal calcium concentration ([Ca^2+^]_i_) to the submicromolar range, and increases both spontaneous and asynchronous release in a calcium-dependent manner. We speculated that Doc2B also recruits Munc13-1 to the PM since these two proteins have been shown to interact physiologically and this interaction is enhanced by Ca^2+^. However, this calcium-dependent co-translocation has never actually been shown. To examine this possibility, we expressed both proteins tagged to fluorescent proteins in PC12 cells and stimulated the cells to investigate the recruitment hypothesis using imaging techniques. We found that Munc13-1 does indeed translocate to the PM upon elevation in [Ca^2+^]_i,_ but only when co-expressed with Doc2B. Interestingly, Munc13-1 co-translocates at a slower rate than Doc2B. Moreover, while Doc2B dislocates from the PM as soon as the [Ca^2+^]_i_ returns to basal levels, Munc13-1 dislocates at a slower rate and a fraction of it accumulates on the PM. This accumulation is more pronounced under subsequent stimulations, suggesting that Munc13-1 accumulation builds up as some other factors accumulate at the PM. Munc13-1 co-translocation and accumulation was reduced when its mutant Munc13-1^H567K^, which is unable to bind DAG, was co-expressed with Doc2B, suggesting that Munc13-1 accumulation depends on DAG levels. These results suggest that Doc2B enables recruitment of Munc13-1 to the PM in a [Ca^2+^]_i_-dependent manner and offers another possible Munc13-1-regulatory mechanism that is both calcium- and Doc2B-dependent.

## Introduction

Munc13-1 is a key player in the final stages of exocytosis. It is a relatively large protein (1,735 aa) with several distinct domains (1). Its Mun domain (aa 859–1531) is responsible for the crucial role of Munc13-1 in exocytosis: the conversion of syntaxin to its open form. This open form of syntaxin interacts with SNAP-25 to form a heterodimer; the heterodimer then interacts with synaptobrevin to form the SNARE complex, which holds the vesicle in close proximity to the plasma membrane (PM) and enables efficient fusion. Munc13-1 has an active C1 domain [aa 567–616 (1, 2)] and has been shown to translocate to the PM upon TPA/PMA stimulation (3). Munc13-1 also has three active C2 domains (4–8), and it interacts with members of the double-C2 domain (Doc2) protein family via residues 851–1461 (3).

Doc2A and B are high-affinity sensors of internal calcium concentration ([Ca^2+^]_i_), containing tandem C2 domains that are responsible for their Ca^2+^-dependent PM targeting and that promote priming and fusion (9, 10). Doc2A interacts with Munc13-1 through its Munc13-interacting domain (Mid, aa 13–37), which is highly conserved in Doc2B [92%, (3)]. The interaction between Munc13 and Doc2 has been demonstrated in both cell-free and intact cell systems. In the yeast two-hybrid system, Munc13-1 and Munc13-2 were shown to interact with both Doc2A and Doc2B (3). Co-immunoprecipitation of Munc13-1 with Doc2A from PC12 cells was markedly enhanced when the cells were stimulated by TPA or high K^+^ in the presence of extracellular calcium (3). A growth hormone assay in PC12 suggested that this interaction has a physiological role; overexpression of the Doc2-interacting domain of Munc13 reduced the Ca^2+^-dependent exocytosis from PC12 cells, and co-expression with Doc2 suppressed this reduction (3). However, expression of Mid alone in PC12 cells had no effect on the number of docked vesicles (11).

The physiological relevance of this interaction was then further tested in neurons. Introduction of the Mid peptide into presynaptic neurons of cholinergic synapses reversibly inhibited synaptic transmission evoked by action potentials (12). In contrast, the scrambled Mid peptide did not inhibit synaptic transmission. Recordings from the calyx of Held revealed that presynaptic loading of a synthetic Mid peptide significantly attenuates phorbol-ester (PE) induced synaptic potentiation, whereas the scrambled Mid peptide had no effect (13).

Imaging experiments revealed that Doc2B translocates to the PM when it is co-expressed with Munc13-1 following PE application (14). Doc2A and Munc13-4 relocated to the cell periphery together with the secretory lysosome marker CD63 upon Ag stimulation in a calcium-free medium (15). This interaction depended on the Mid domain of Doc2A although C2B also seemed to play a role (15). Both Doc2B and Munc13-1 are expressed in chromaffin cells (10, 16). Based on the fact that Doc2B translocates to the PM upon calcium elevation and interacts with Munc13-1, and on the above data, we hypothesized that Doc2B will efficiently recruit Munc13-1 to the PM in a [Ca^2+^]_i_-dependent manner. We found that Munc13-1 co-translocates to and accumulates at the PM when co-expressed with Doc2B in a calcium-dependent manner.

## Materials and Methods

### Cell lines

PC12 cells (a generous gift from Dr. Nicolas Vitale, CNRS-UPR, Strasbourg, France) were grown in DMEM (Biological Industries, Beit Haemek, Israel) supplemented with glucose (4,500 mg/l) and l-glutamine (Gibco-BRL), and containing 5% (v/v) fetal calf serum, 10% (v/v) horse serum, and 100 U/ml penicillin/streptomycin (Biological Industries). The cells were split regularly to maintain confluence and were kept in a 37°C, 5% CO_2_-humidified incubator. Transfection was performed using Lipofectamine™ 2000 (Invitrogen) according to the manufacturer’s instructions at a 2:3 (DNA:reagent) ratio. Cells were imaged 16–32 h after transfection.

COS-7 cells were grown in DMEM supplemented with 10% fetal calf serum, 2 mM l-glutamine, and 100 U/ml penicillin-streptomycin. The cells were split regularly to maintain confluence and were kept in a 37°C, 5% CO_2_-humidified incubator. Transfection was performed using Jet-PEI (PolyPlus Transfection, New York, NY, USA) according to the manufacturer’s instructions at a 1:2 (DNA:reagent) ratio.

### DNA constructs

All plasmids encoding fluorescently labeled Doc2B and the Doc2B–glutathione-*S*-transferase (GST) plasmids were a generous gift from Dr. Alexander J. Groffen (Vrije Universiteit, Amsterdam, The Netherlands) and the control GST construct was a generous gift from Prof. Joel Hirsch (Tel Aviv University, Israel). The sequences of all constructs were verified by automated DNA sequencing. Munc13-1 plasmids are a kind gift from Prof. N. Brose (Max-Plank institute, Gottingen, Germany).

### Western blotting

Western blot experiments were performed according to standard procedures. In general, protein extracts (for overexpression experiments ∼10 μg protein, for endogenous experiments ∼100 μg protein) were loaded on an SDS-polyacrylamide (8 or 11%) gel and electrophoresed with a constant current of 30 mA for each gel. The proteins from the gel were transferred to a nitrocellulose membrane by electroblotting at a constant current of 400 mA for 1 h. The membrane was incubated in blocking solution (5% milk powder) overnight at 4°C with gentle agitation. After five washes, we incubated the membrane with primary rabbit anti-Doc2B antibody for 1 h at room temperature (diluted 1:500 in 1% BSA with 0.05% azide). The membrane was washed five times and incubated with the relevant secondary horseradish peroxidase-conjugated antibody for 45 min at room temperature (diluted 1:15,000 in milk). Then the membrane was washed six times, and detection was performed using enhanced chemiluminescence solution (Pierce) and exposure to Super RX film (Fuji).

### Co-translocation experiments

For the translocation experiment, PC12 cells were plated on glass coverslips, transfected with different combinations of plasmids (see Results for details) encoding fluorescently tagged proteins – Doc2B^wt^–mRFP, Doc2B^D218,220N^–mRFP, Doc2B^Mid^–mRFP, Munc13-1^wt^–EGFP, Munc13-1^H567K^–EGFP, or EGFP alone using Lipofectamine 2000. Imaging was performed 16–32 h post-transfection. Cells were perfused constantly with external solution and excited using a high K^+^ solution (70 mM) as described in Groffen et al. (9). The imaging setup consisted of an Olympus IX-70 inverted microscope with a 60× total internal reflection fluorescence (TIRF) objective (Olympus), a TILL Photonics TIRF condenser (Gräfelfing, Germany), two solid-state lasers (Laser Quantum, Stockport, UK) emitting at 473 and 532 nm, an Andor Ixon 887 EMCCD camera (Belfast, Northern Ireland), and a dual-view beam-splitting device (Optical Insights, Roper Bioscience, Tucson, AZ, USA). Time-lapse images were taken every 300 ms. The equipment was controlled by Metamorph software (Molecular Devices, Downingtown, PA, USA), which was also used to perform the analysis. Confocal images were recorded using a 63× objective of Zeiss LSM 510 META microscope equipped with 30 mW 488 nm Argon laser and 15 mW 561 nm DPSS laser.

## Results

### Doc2B and Munc13-1 co-translocate upon elevation of [Ca^2+^]_i_

**Figure 1:**
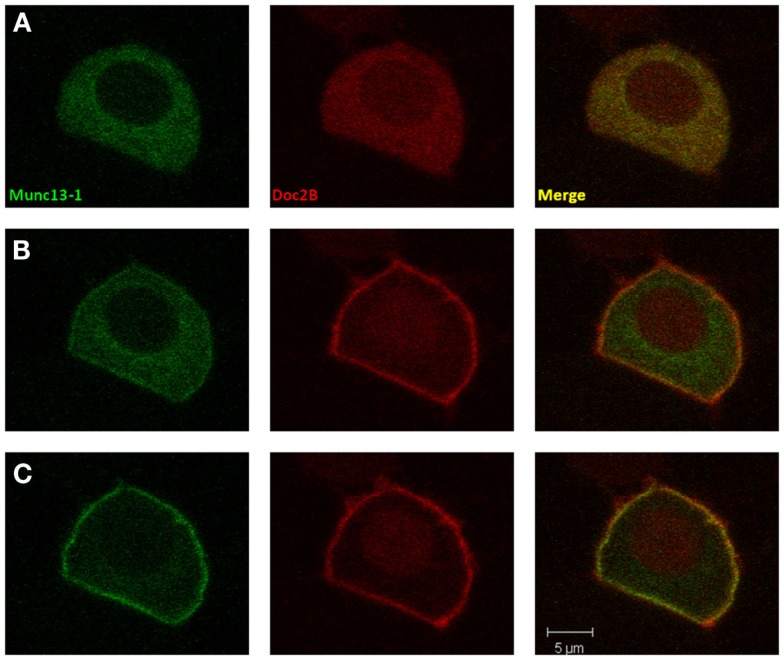
**Co-translocation of Doc2B^wt^ and Munc13-1^wt^**. Confocal images of a PC12 cell co-expressing Munc13-1^wt^-EGFP (left) and Doc2B^wt^-mRFP (middle). Merged images presented on the right. **(A)** At basal calcium both proteins have a cytoplasmic distribution. **(B)** At the initial phase of the calcium elevation (2–10 s after KCl application), translocation of Doc2B occurs, but Munc13-1 is still mainly cytoplasmic. **(C)** Munc13-1^wt^ translocation is seen only at later stages of the experiment when Doc2B concentration on the membrane is higher (10–20 s after KCl application).

**Figure 2:**
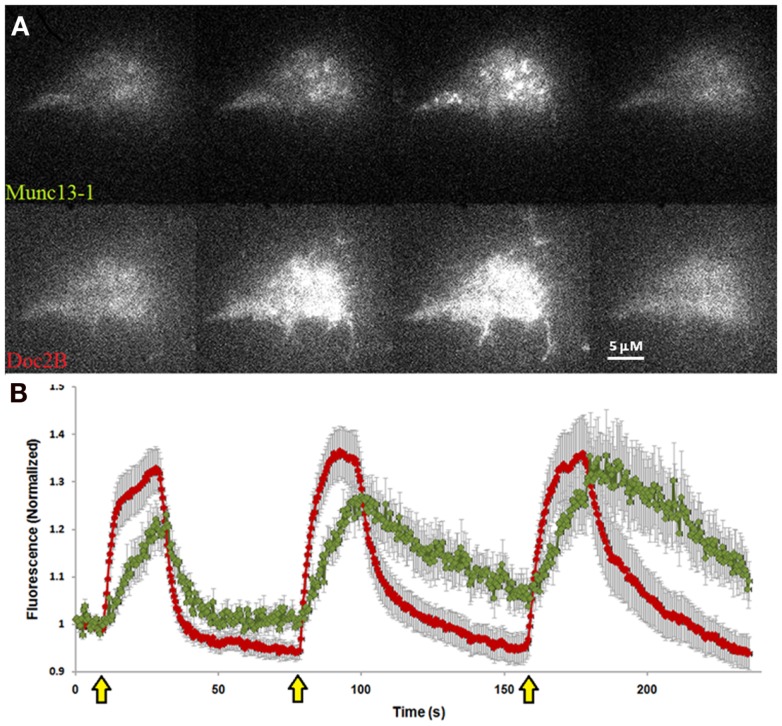
**Co-translocation of Doc2B^wt^–mRFP and Munc13-1^wt^–EGFP in the TIRF plane**. **(A)** TIRF images of a PC12 cell expressing Munc13-1^wt^–EGFP (upper panel) and Doc2B^wt^–mRFP (lower panel). From left to right: basal state, beginning of translocation, maximum translocation, and back to basal state. **(B)** Quantification graph of the translocation kinetics observed in the cell’s lower PM. Red line indicates Doc2B^wt^–mRFP, green line indicates Munc13-1^wt^–EGFP (*n* = 13). Yellow arrows indicate KCl application.

**Figure 3:**
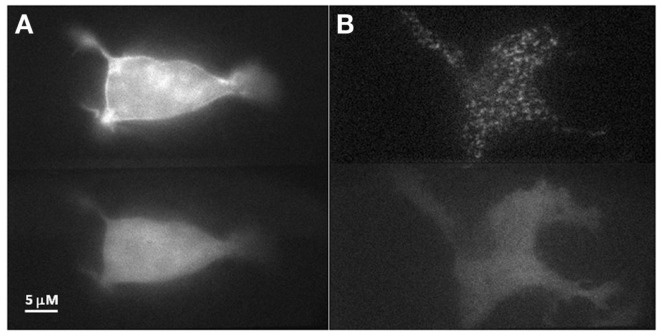
**PE stimulation of PC12 cell co-expressing Doc2B^wt^–mRFP and Munc13-1^wt^–EGFP**. **(A)** Epi-fluorescence images of a PC12 cell co-expressing Munc13-1^wt^–EGFP (upper panel) and Doc2B^wt^–mRFP (lower panel) after PE application. Munc13-1^wt^ translocated to the PM while Doc2B^wt^ did not. **(B)** TIRF images of a PC12 cell co-expressing Munc13-1^wt^–EGFP (upper panel) and Doc2B^wt^–mRFP (lower panel) after PE application. Munc13-1^wt^ translocated to the PM while Doc2B^wt^ did not. Note the patchy nature of Munc13-1^wt^ ’s translocation at the PM.

To investigate our hypothesis that co-expression of Doc2B^wt^ and Munc13-1 results in co-translocation of both proteins to the PM upon [Ca^2+^]_i_ elevation, we expressed Munc13-1 fused to EGFP in PC12 cells together with Doc2B^wt^ fused to mRFP. The cells were subjected to three short (20 s) KCl applications (with relaxation times between each application) which caused elevation of [Ca^2+^]_i_ and translocation of Doc2B and Munc13-1 to the PM (Figure 1). In the absence of calcium, the translocation of both proteins was abolished suggesting it is calcium dependent (Figure S1 in Supplementary Material). Munc13-1 translocated to the PM upon elevation in [Ca^2+^]_i_, but only when co-expressed with Doc2B^wt^ (Figures 1 and 2). When Munc13-1 was expressed in PC12 cells without Doc2B it did not translocate to the PM upon high K^+^ stimulation (Figure S2 in Supplementary Material). To establish a more quantitative connection between Doc2B^wt^ translocation and that of Munc13-1^wt^, we repeated this experimental protocol using TIRF microscopy, focusing on the fluorescence of the cell’s lower PM (Figure 2A). Co-translocation of Munc13-1^wt^ and Doc2B^wt^ was clearly seen in the TIRF plane (Figure 2A). Examining the translocation kinetics revealed that Munc13-1^wt^ translocates to the PM at a slower rate than Doc2B^wt^ (Figure 2B; *n* = 13). Moreover, Munc13-1^wt^ translocation, at least in the second and third application, peaked when Doc2B^wt^ was already in its dislocating phase (Figure 2B). Munc13-1 dislocation also seemed to occur at a slower rate than that of Doc2B. It is interesting to note that although Doc2B^wt^ fluorescence returned to its initial level after each application, Munc13-1^wt^ started to accumulate at the PM after the first application, though this became more apparent after the second and third application. It is also interesting to note that although the translocation of Munc13^wt^ to the PM appeared rather uniform in the confocal images (Figure 1), it showed a patchy appearance in TIRF images (Figure 2A). This patchy pattern was not unique to Munc13-1^wt^’s Doc2B^wt^-dependent translocation; a similar dotted staining pattern appeared in PE-induced translocation of Munc13-1^wt^ (Figure 3). As PE mimics the interaction of Munc13-1 with diacylglycerol (DAG), it is possible that when on the PM, Munc13-1 interacts with DAG.

**Figure 4:**
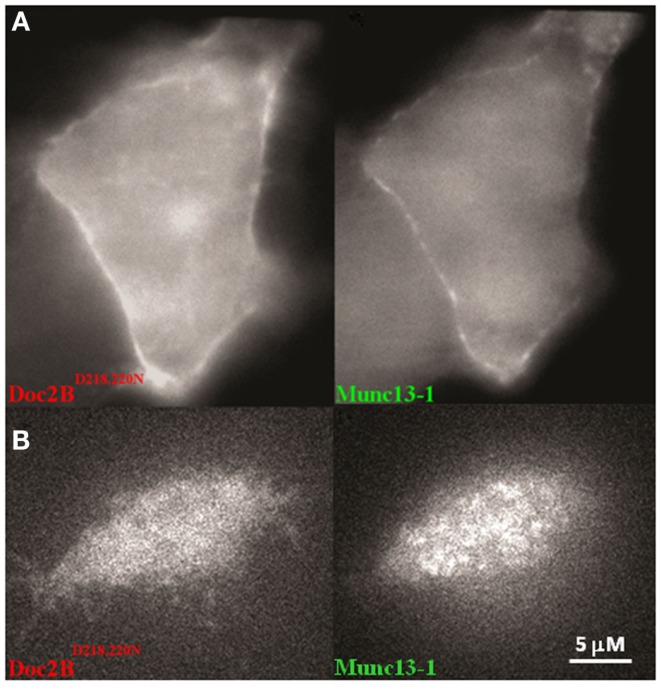
**A PC12 cell co-expressing Doc2B^D218,220N^–mRFP and Munc13-1^wt^**. **(A)** Epi-fluorescence images of a PC12 cell co-expressing Doc2B^D218,220N^–mRFP (left) and Munc13-1^wt^–EGFP (right). **(B)** TIRF images of a PC12 cell co-expressing Doc2B^D218,220N^–mRFP (left) and Munc13-1^wt^–EGFP (right).

We have previously shown that a mutated form of Doc2B–Doc2B^D218,220N^ is constitutively associated with the PM. We therefore examined the effect of Doc2B^D218,220N^ on Munc13-1^wt^’s distribution in the cell. Co-expression of these two proteins resulted in the constant translocation of both Doc2B^D218,220N^ and Munc13-1^wt^ (Figure 4). As in Figure 2, Munc13-1^wt^ showed a patchy pattern at the PM.

### Co-translocation of Munc13-1 and Doc2B depends on the Doc2B–Munc13-1 interaction and on Munc13-1s C1 domain

**Figure 5:**
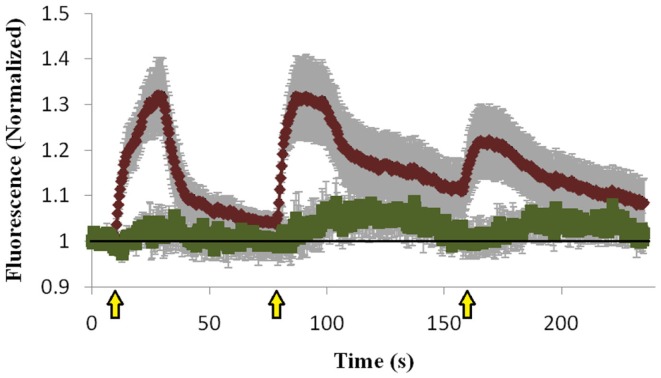
**Quantification of co-translocation of Doc2B^Mid^–mRFP and Munc13-1^wt^–EGFP**. Quantification graph of the TIRF experiment demonstrating translocation of Doc2B^Mid^ and low translocation of Munc13-1^wt^ during the first KCl application (*n* = 8). Munc13-1^wt^ translocation becomes more pronounced at the second and third KCl application but still lower compared to co-expression with Doc2B^wt^. Black line marks the value of 1 (no translocation). Yellow arrows indicate KCl application.

To determine whether the Mid domain of Doc2B is responsible for the co-translocation of Doc2B and Munc13-1, we repeated the translocation experiment with Doc2B harboring a scrambled Mid domain (Doc2B^Mid^), and examined if this mutation abolishes translocation of Munc13-1^wt^. Scrambling the Mid domain of Doc2B disrupted the interaction with Munc13-1^wt^ [Figure S3 in Supplementary Material; (12)]. Although the Mid mutation disrupted most of Munc13-1^wt^’s translocation, some degree of translocation still existed (Figure 5). The translocation was barely detected in the first KCl application but in later applications, some Munc13-1 showed translocation and accumulation on the PM. These results suggest that the Mid domain is important for Munc13-1 co-translocation; however, Munc13-1 also accumulates slowly at the PM without the interaction with Doc2B Mid domain, possibly via its C1–DAG interaction.

**Figure 6:**
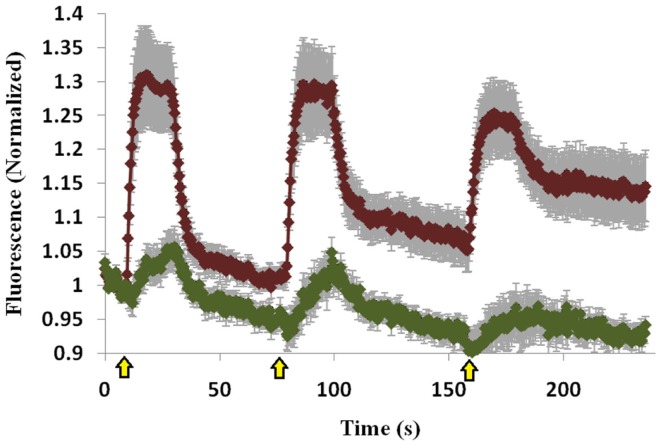
**Co-translocation of Doc2B^wt^–mRFP and Munc13-1^H567K^–EGFP**. Quantification graph of the TIRF experiment demonstrating translocation of Doc2B^wt^ and low translocation of Munc13-1^H567K^ (*n* = 14). No accumulation of Munc13-1^H567K^ is detected. Yellow arrows indicate KCl application.

The C1 domain of Munc13 is important for its membrane-attachment ability via its interaction with DAG (17, 18), and we therefore examined whether the C1 domain is also important for Doc2B-induced Munc13-1 translocation and accumulation. We used a Munc13-1 mutant that does not bind DAG (Munc13-1^H567K^) and cannot translocate to the PM upon PE stimulation, and examined whether Doc2B could induce Munc13-1^H567K^ translocation. Munc13-1^H567K^ displayed lower translocation ability than Munc13-1^wt^ (Figure 6 compared to Figure 2) and did not accumulate on the PM like its wild-type counterpart. Thus, it seems that the initial translocation of Munc13-1 depends on interaction with Doc2B and soon after, the C1–DAG interaction determines the accumulation of Munc13-1 at the PM.

## Discussion

Munc13-1 is a key player in the synapse; the activity of this multidomain protein is tightly regulated by many factors, including calmodulin (5, 19, 20), DAG (18, 21), and Ca^2+^ in a PIP_2_ dependent manner (7). We show here that Doc2B recruits Munc13-1 to the PM in a Ca^2+^-dependent manner, thereby suggesting another possible Munc13-1-regulatory mechanism.

The Doc2 family of proteins interacts with the Munc13 family of proteins primarily via the Mid domain, located within the N-terminal domain of Doc2 (3, 12, 14, 15, 22). Doc2 translocates to the PM upon elevation of [Ca^2+^]_i_. Munc13-1, on the other hand, does not translocate to the PM upon calcium elevation. However, when co-expressed with Doc2B, Munc13-1 co-translocates to the PM following Doc2B. This translocation could not be detected with endogenous Doc2B and Munc13-1 as these proteins are expressed in low levels in neuroendocrine cells (10, 16, 19) and such translocation, if occurs might be undetectable under these conditions. Munc13-1’s translocation is most likely mediated through the Mid domain as reflected by its reduction upon mutation in the Mid domain. When the elevation of calcium is brief, Munc13-1 translocation is reversible, dislocating back to the cytosol after Doc2B dislocation. However, following repeated stimulations, Munc13-1 starts to accumulate at the PM even after Doc2B has dislocated back to the cytosol. This accumulation was abolished when a Munc13-1 mutant that does not bind DAG (Munc13-1^H567K^) was co-expressed with Doc2B suggesting that the C1–DAG interaction determines Munc13-1’s accumulation at the PM. It is possible that during high-frequency activity, e.g., sustained or intermittent depolarization, the level of DAG increases (23). This would cause a more stable interaction of Munc13-1 with the PM via its C1 domain, anchoring it to the PM, and enabling its catalytic activity in the fusion step (2). Thus, according to this working model, the activity of Munc13-1 at the PM depends on the stimulation’s frequency and on Doc2B translocation, becoming more prominent during periods of high activity. Doc2B has been recently shown to enhance neuronal network activity by specifically increasing the firing rate within a neuronal burst (24). Therefore, it is possible that in addition to the direct effect of Doc2B on asynchronous release, it also recruits Munc13-1 during periods of intense activity, which might also contribute to the enhanced network activity. A similar mode of action has been suggested for protein kinase C (PKC) activity (25). Many receptor stimuli induce calcium signals prior to a more persistent increase in DAG concentration. These calcium signals have only a minimal effect on conventional PKC activity in the absence of DAG. However, in the presence of DAG, each calcium spike induces a more pronounced activation cycle of conventional PKC.

Our data suggest that the interaction between Doc2B and Munc13-1 depends on more than just the Mid domain of Doc2B since a small degree of translocation was still observed when Munc13-1 was co-expressed with Doc2B^Mid^. We hypothesize that this interaction is also dependent on the C1 domain of Munc13-1 as it has been reported that deleting this domain increases Munc13-1’s interaction with Doc2B (3). It is also possible that the C2B of Doc2B contributes to this interaction, as has been recently suggested for Doc2A and Munc13-4 (15).

A previous study described co-translocation of Doc2B^wt^ and Munc13-1^wt^ upon PE stimulation (14). Such translocation was not observed here, either in the epi-fluorescence or TIRF imaging. Hence, the interaction of Munc13-1’s C1 domain with DAG at the PM may compete with Doc2B binding to Munc13-1. Therefore, stimulating the cells with PE caused Munc13-1’s translocation but interfered with Doc2B translocation. These findings, together with the observation that Munc13-1 reaches maximal translocation when Doc2B is already dislocating from the PM due to a decrease in [Ca^2+^]_i_, suggest that the interaction of Munc13-1 with DAG at the PM cannot occur when it is in a complex with Doc2B. Therefore, DAG binding to Munc13-1 might disrupt Doc2B binding. These findings suggest that Doc2B recruits Munc13-1 to the PM but once there, Munc13-1 associates through its C1 domain with DAG and this reduces its interaction with Doc2B. Further experiments will be needed to validate this hypothesis.

Co-expression of Doc2B^D218,220N^ with Munc13-1^wt^ revealed that the two proteins are being constantly translocated to the PM, yet the Doc2B^D218,220N^ mutant could not support refilling during repeated stimulations (10). Combining these two observations suggests a possible physiological effect of the Doc2B–Munc13-1 interaction: this complex needs to undergo an on–off cycle from the PM to achieve a full priming effect. Another possible explanation is that Munc13-1 needs to detach from Doc2B at the PM to enable its priming effect. Taken together, our data support the hypothesis that Doc2B serves as a calcium-dependent recruitment factor for Munc13-1 whereas at the PM, Munc13-1 interacts with DAG. This provides an activity-dependent recruitment mechanism for two major synaptic proteins, Doc2B and Munc13-1.

## Conflict of Interest Statement

The authors declare that the research was conducted in the absence of any commercial or financial relationships that could be construed as a potential conflict of interest.

## Supplementary Material

The Supplementary Material for this article can be found online at: http://www.frontiersin.org/Neuroendocrine_Science/10.3389/fendo.2013.00119/abstract

Figure S1 **Doc2B^wt^ and Munc13-1^wt^ do not translocate to the PM in the absence of calcium**. Epi-fluorescence images of a PC12 cell co-expressing Doc2B^wt^-mRFP (left) and Munc13-1^wt^-EGFP (center). Merged images presented on the right. In the upper panel, the cell in its basal state. In the lower panel, the cell after application of depolarizing high K^+^ solution without calcium (containing 0.1 mM EGTA). Note there is no evident change in the proteins distribution in the cell.

Figure S2 **Munc13-1^wt^ does not translocate to the PM in the absence of Doc2B**. Epi-fluorescence images of a PC12 cell expressing Munc13-1^wt^–EGFP, before (left) and after (right) application of depolarizing high K^+^ solution. Note there is no evident change in the protein’s distribution in the cell.

Figure S3 **GST pull-down assay of Doc2B N-terminal with Munc13-1**. GST fusion of the N-terminal of Doc2B^wt^ and N-terminal of Doc2B^Mid^ binding to Munc13-1^wt^ (Upper panel) and Munc13-1^H567K^ (lower panel). Munc13-1^wt^ and Munc13-1^H567K^ show binding only to N-terminal of Doc2B^wt^ and not to N-terminal of Doc2B^Mid^.
