# Supplementary material for: Munc13-1 Translocates to the Plasma Membrane in a Doc2B- and Calcium-Dependent Manner
Source: Front Endocrinol (Lausanne). 2013 Sep 17;4:119. doi: 10.3389/fendo.2013.00119 (PMC3775473; doi:10.3389/fendo.2013.00119)
Supplement: Figure S1 — Doc2Bwt and Munc13-1wt do not translocate to the PM in the absence of calcium. Epi-fluorescence images of a PC12 cell co-expressing Doc2Bwt-mRFP (left) and Munc13-1wt-EGFP (center). Merged images presented on the right. In the upper panel, the cell in its basal state. In the lower panel, the cell after application of depolarizing high K+ solution without calcium (containing 0.1 mM EGTA). Note there is no evident change in the proteins distribution in the cell. [file 59450_Ashery_DataSheet1.DOC]

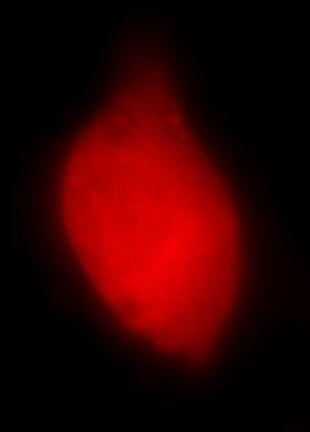

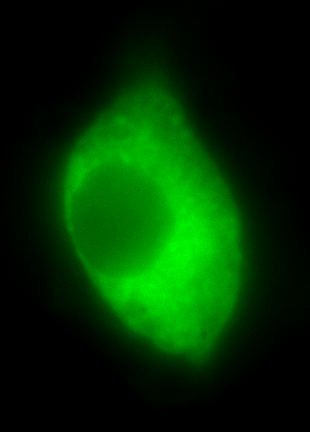

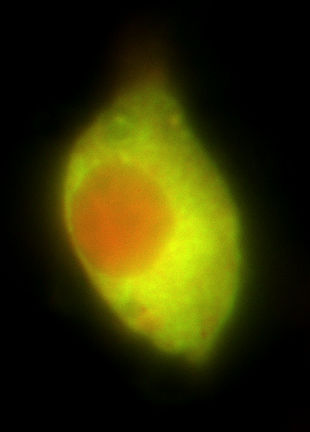

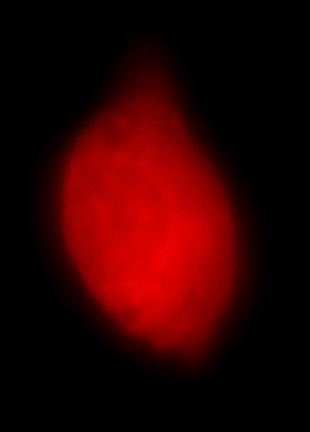

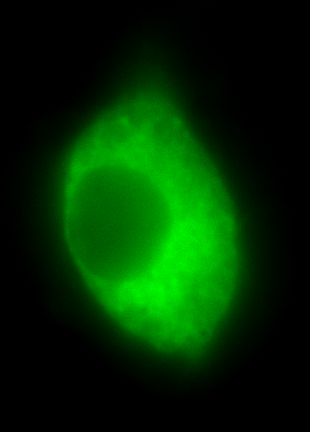


**Munc13-1**

**Doc2B**

**Merge**

5 μm

**Figure 1S Doc2B^wt^ and Munc13-1^wt^ do not translocate to the PM in the absence of calcium.** Epi-fluorescence images of a PC12 cell co-expressing Doc2B^wt^-mRFP (left) and Munc13-1^wt^-EGFP (center). Merged images presented on the right. In the upper panel, the cell in its basal state. In the lower panel, the cell after application of depolarizing high K^+^ solution without calcium (containing 0.1mM EGTA). Note there is no evident change in the proteins distribution in the cell.


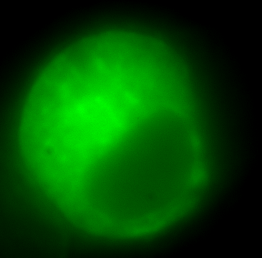


5 μm

**Figure 2S Munc13-1^wt^ does not translocate to the PM in the absence of Doc2B.** Epi-fluorescence images of a PC12 cell expressing Munc13-1^wt^-EGFP, before (left) and after (right) application of depolarizing high K^+^ solution. Note there is no evident change in the protein's distribution in the cell.


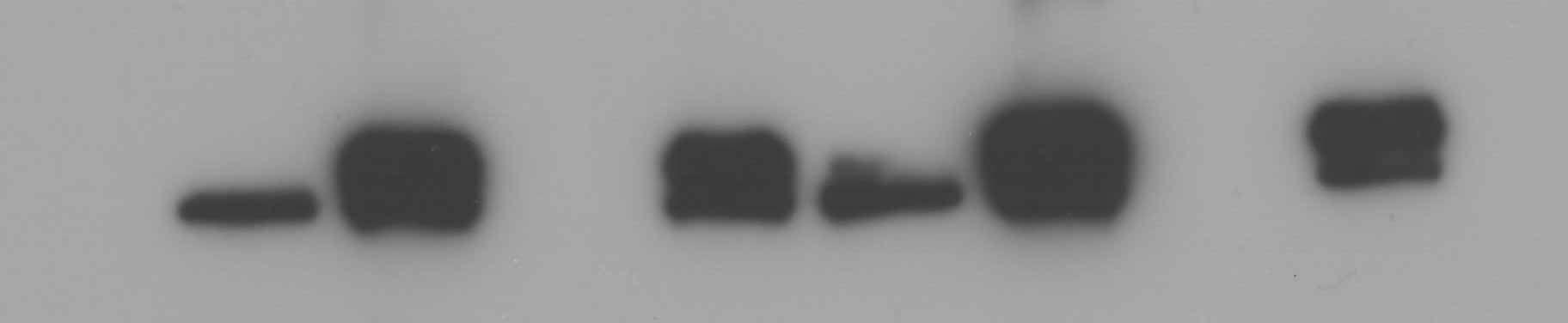

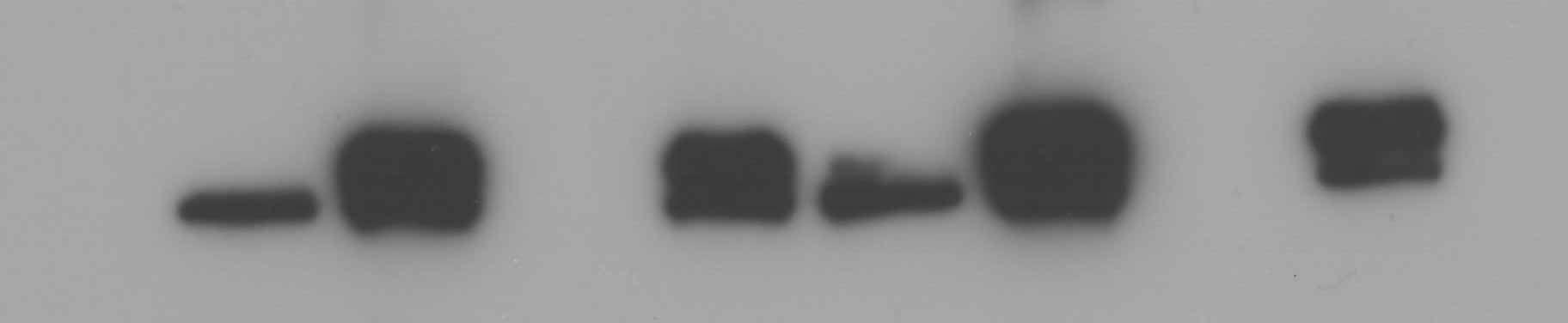


**N-Term Doc2B^Mid^**

**N-Term Doc2B^WT^**

**Munc13-1^WT^**

**Munc13-1^H567K^**

**Figure 3S GST pull-down assay of Doc2B N-Terminal–Munc13-1**. GST fusion of the N-terminal of Doc2B^wt^ and N-terminal of Doc2B^Mid^ binding to Munc13-1^wt^ (Upper panel) and Munc13-1^H567K^ (lower panel). Munc13-1^wt^ and Munc13-1^H567K^ show binding only to N-terminal of Doc2B^wt^ and not to N-terminal of Doc2B^Mid^.

**Supplemental Methods**

### GST pull-down

The N terminus of Doc2B^wt^ (aa 1–126) and the N terminus of Doc2B^Mid^ (aa 1–126 with scrambled Mid domain) were expressed in *Escherichia coli* strain td+ as GST-fusion proteins. Control GST was expressed in a similar manner. When the bacteria had grown to an OD of 0.6, IPTG was added to a final concentration of 250 μM. When the OD exceeded 0.9, the bacteria were harvested in cold STE buffer, consisting of (in mM): 10 Tris pH 8, 150 NaCl, 1 EDTA. Lysozyme was added to a final concentration of 100 μg/ml and the supernatant was incubated on ice for 15 min. 5 mM DTT and 1.5% n-octylglucoside were added. The homogenate was sonicated on ice for 1 min and centrifuged at 10,000*g* for 5 min at 4ºC. Triton X-100 was added to the supernatant to a final concentration of 2%. The samples were vortexed, prepared glutathione–agarose beads were added, and the mixture was incubated for 15 min on a shaking device at 4ºC. Then the samples were centrifuged (5000 rpm, 5 min, 4ºC) and washed with cold phosphate buffered saline (PBS), four times. After the last centrifugation, the pellet was resuspended in storage buffer containing (in mM) 50 HEPES pH 7.5, 150 NaCl, 5 DTT, 10% glycerol and protease inhibitor cocktail (Calbiochem, set I). The beads were subjected to SDS-PAGE as above and the membrane was reacted with rabbit anti-Doc2B antibody for verification. EGFP-tagged Munc13-1^wt^ and Munc13-1^H567K^ were expressed in COS-7 cells by Jet-PEI transfection. Lysates were prepared from the cells 34–36 h after transfection using solubilization buffer containing (in mM): 150 NaCl, 50 HEPES pH 7, 1.5 MgCl_2_, 1 mM EDTA, 1 mM EGTA, 0.5 mM DTT, 10% glycerol, 1% Triton X-100, and protease inhibitor cocktail (Calbiochem, set I), and incubated on ice for 10 min. Then the homogenates were centrifuged (10 min 14,000 rpm, 4ºC) to eliminate the debris and protein amount in the supernatants was estimated by Bradford assay. A 500-μg aliquot of the protein was taken for each reaction together with 70 μl of beads and incubated for 30 min on a shaking device at 4ºC. The product was washed three times (14,000 rpm, 2 min, 4ºC) with solubilization buffer. After the last centrifugation, sample buffer was added to the beads and they were vortexed thoroughly. The samples were boiled at 100ºC for 5 min, vortexed and centrifuged at room temperature for 5 min. The supernatant was subjected to SDS-PAGE as described above and immunoblotting with anti-Munc13-1 (a generous gift from Dr. Varoqueaux, Max Planck Institute, Gottingen Germany, diluted 1:500) or with anti-GFP (BD Biosciences, San Diego, CA).
